# Association of Mechanical Bowel Preparation and Oral Antibiotics Before Elective Colorectal Surgery With Surgical Site Infection: A Network Meta-analysis

**DOI:** 10.1001/jamanetworkopen.2018.3226

**Published:** 2018-10-12

**Authors:** James W. T. Toh, Kevin Phan, Kerry Hitos, Nimalan Pathma-Nathan, Toufic El-Khoury, Arthur J. Richardson, Gary Morgan, Alexander Engel, Grahame Ctercteko

**Affiliations:** 1 Discipline of Surgery, Sydney Medical School, The University of Sydney, Sydney, Australia; 2 Westmead Research Centre for Evaluation of Surgical Outcomes, Department of Surgery, Westmead Hospital, Sydney, Australia; 3 Division of Surgery and Anaesthetics, Department of Surgery, Westmead Hospital, Sydney, Australia; 4 University of Notre Dame, Sydney, Australia; 5 Department of Surgery, Royal North Shore Hospital, Sydney, Australia

## Abstract

**Importance:**

There has been a resurgence of interest in the use of mechanical bowel preparation (MBP) and oral antibiotics (OAB) before elective colorectal surgery. Until now, clinical trials and meta-analyses have not compared all 4 approaches (MBP with OAB, OAB only, MBP only, or no preparation) simultaneously.

**Objective:**

To perform a network meta-analysis to clarify which approach in colorectal surgery is associated with the lowest rate of surgical site infection (SSI).

**Data Sources:**

Five electronic databases were searched, including PubMed, Cochrane Central Register of Controlled Trials, Cochrane Database of Systematic Reviews, ACP Journal Club. and Database of Abstracts of Review of Effectiveness from database inception to November 27, 2017.

**Study Selection:**

Only data from randomized clinical trials were included. Inclusion criteria were RCTs that reported on SSI rates or other complications based on MBP or OAB status. Quality of studies was appraised by the Cochrane Collaboration risk of bias tool.

**Data Extraction and Synthesis:**

The study was performed according to the Preferred Reporting Items for Systematic Reviews and Meta-Analyses (PRISMA) guidelines.

**Main Outcomes and Measures:**

Total, incisional, and organ/space SSI rates. Secondary outcomes included rates of anastomotic leak, mortality, readmissions/reoperations, urinary tract infection, and pulmonary complications.

**Results:**

Thirty-eight randomized clinical trials among 8458 patients (52.1% male) were included, providing 4 direct comparisons and 2 indirect comparisons for 8 outcome measures. On Bayesian analysis, MBP with OAB vs MBP only was associated with reduced SSI (odds ratio [OR], 0.71; 95% equal-tail credible interval [CrI], 0.57-0.88). There was no significant difference between MBP with OAB vs OAB only (OR, 0.95; 95% CrI, 0.56-1.62). Oral antibiotics without MBP was not associated with a statistically significant reduction in SSI compared with any other group (except for a risk reduction in organ/space SSI when indirectly compared with no preparation) (OR, 0.13; 95% CrI, 0.02-0.55). There was no difference in SSI between MBP only vs no preparation (OR, 0.84; 95% CrI, 0.69-1.02).

**Conclusions and Relevance:**

In this network meta-analysis of randomized clinical trials, MBP with OAB was associated with the lowest risk of SSI. Oral antibiotics only was ranked as second best, but the data available on this approach were limited. There was no difference between MBP only vs no preparation. In addition, there was no difference in rates of anastomotic leak, readmissions, or reoperations between any groups.

## Key Points

**Question:**

Which approach is best to reduce surgical site infections and anastomotic leak in colorectal surgery: mechanical bowel preparation with oral antibiotics, oral antibiotics only, mechanical bowel preparation only, or no preparation?

**Findings:**

Among 38 randomized clinical trials (8458 patients) in this network meta-analysis, mechanical bowel preparation with oral antibiotics was associated with the lowest rate of surgical site infections, reducing both incisional and organ/space infections. There was no significant difference in anastomotic leak rate between the 4 approaches.

**Meaning:**

Mechanical bowel preparation with oral antibiotics is the best approach to reduce surgical site infections in patients undergoing colorectal surgery.

## Teaser

This network meta-analysis of randomized clinical trials assesses which approach in colorectal surgery (mechanical bowel preparation with and without oral antibiotics) is associated with the lowest rate of surgical site infections.

## Introduction

There has been a resurgence of interest in the use of mechanical bowel preparation (MBP) and oral antibiotics (OAB) before elective colorectal surgery (CRS). This is a current and controversial topic, not owing to a lack of high-quality evidence but because studies on this topic have reported a diverse range of outcomes.

Despite the American Society for Enhanced Recovery and the Perioperative Quality Initiative joint consensus statement recommending the “routine use of a combined isosmotic mechanical bowel preparation with oral antibiotics before elective CRS”^1^^(p6)^ to reduce surgical site infection (SSI), there has been a lack of consensus between international guidelines in United States, Europe, and Asia-Pacific. The recent Australian guidelines recommended that “mechanical bowel preparation should not be used routinely in colonic surgery.”^2^

Furthermore, a 2017 survey of European colorectal surgeons found that few European surgeons used OAB despite recent evidence suggesting that preoperative OAB reduced SSI.^3^ Less than 10% of the European colorectal surgeons who participated in the survey prescribed preoperative OAB with perioperative intravenous antibiotics (IVAB), with 96% choosing to prescribe perioperative IVAB only. Most also indicated that they used MBP before rectal surgery, and 30% used MBP before colonic surgery^3^ despite a large body of evidence showing no benefit with MBP.^4^

Several meta-analyses^4,5,6,7,8^ have already been published on MBP and OAB before CRS. However, traditional meta-analysis techniques have only provided comparisons between 2 approaches (MBP vs no preparation, OAB vs no preparation, MBP with OAB vs no preparation, and MBP with OAB vs OAB). Therefore, it has been difficult to compare the results of studies that have reported on different permutations of MBP and OAB regimens. To compare all 4 approaches simultaneously (MBP with OAB, OAB only, MBP only, or no preparation), we performed the first network meta-analysis (NMA) to date, to our knowledge, of randomized clinical trials (RCTs) only on this topic.

The advantage of performing an NMA over traditional meta-analysis on this topic was the ability to compare all 4 treatment arms of trials that assessed MBP and OAB. As a result, we were able to rank all 4 MBP and OAB regimens simultaneously, facilitating side-by-side comparisons of the 4 approaches. By comparing multiple treatment arms using only evidence from RCTs, this NMA synthesizes and clarifies the existing evidence on MBP and OAB before elective CRS.

## Methods

### Literature Search Strategy

The present study was performed according to the Preferred Reporting Items for Systematic Reviews and Meta-Analyses (PRISMA) reporting guideline^9,10^ (eFigure 1 in the Supplement). Five electronic databases were searched, including PubMed, Cochrane Central Register of Controlled Trials, Cochrane Database of Systematic Reviews, ACP Journal Club, and Database of Abstracts of Review of Effectiveness from database inception to November 27, 2017. To minimize the risk of overlooking relevant studies and given the wide variety of procedural nomenclature, it was necessary to combine a large number of keywords and Medical Subject Headings, resulting in the following search terms: *mechanical bowel preparation*, *oral antibiotics*, *colon*, *rectal*, *colorectal*, and *surgery*. Moreover, reference lists of relevant literature were examined for any further studies. Literature search strategy, selection process, data extraction, and assessment of quality of studies are reported in the eAppendix in the Supplement.

### Inclusion and Exclusion Criteria

Inclusion criteria were RCTs that reported on SSI rates or other complications based on MBP or OAB status. When institutions published subsequent studies with accumulating numbers of patients or increased lengths of follow-up, only the most complete reports were included for quantitative assessment at each interval. Exceptions to this protocol were studies by Zmora et al^11^ in 2003 and by Zmora et al^12^ in 2006, both of which reported on MBP with OAB vs OAB only. The 2006 study^12^ reported on left-sided CRS, whereas the 2003 study^11^ reported on both sides. The 2003 study^11^ recruited more patients, but the 2006 study^12^ was included in this NMA because it provided good qualitative data on the role of MBP and OAB in left-sided colonic surgery and because the direct comparison between MBP with OAB vs OAB only had limited data.

Studies^13,14^ involving the use of the older oral antiprotozoan antibiotic tinidazole were excluded from analysis. Tinidazole is commonly used to treat protozoan, amebic, and parasitic infections.

All publications were limited to those involving human participants and in the English language. Abstracts, case reports, conference presentations, editorials, and expert opinions were excluded. Review articles were omitted because of potential publication bias and duplication of the results.

### Data Extraction and Quality Assessment of Studies

Two reviewers (J.W.T.T. and K.P.) independently reviewed and appraised studies using a standard form and extracted data on methods and outcome measures. Discrepancies between the 2 reviewers were resolved by discussion and consensus. In addition, quality of studies was appraised by the Cochrane Collaboration risk of bias tool (version 5.0.1) (eTable 1 in the Supplement). This tool included the following components: selection bias (defined as random sequence generation and allocation concealment), performance bias (masking of both participants and investigators), detection bias (masking of evaluators), attrition bias (incomplete outcome data), and reporting bias (selective outcome reporting). Each component was judged to be of low, unclear, or high risk of bias. Discrepancies between reviewers were resolved by discussion and consensus.

### Outcome Assessment

The primary outcome measures were total, incisional, and organ/space SSI rates as defined by the US Centers for Disease Control and Prevention.^15^ Secondary outcomes included rates of anastomotic leak, mortality, readmissions/reoperations, urinary tract infection (UTI), and pulmonary complications.

### Statistical Analysis

We conducted an NMA using a Bayesian Markov chain Monte Carlo method in WinBUGS 1.4.3 (MRC Biostatistics Unit, Cambridge, United Kingdom) through the conduit of the Microsoft Excel–based macro NetMetaXL 1.6.1 (Canadian Agency for Drugs and Technologies in Health).^16^ A convergence test for each analysis was conducted by checking whether the Monte Carlo error was less than 5% of the SD of the effect estimates or the variance between the studies. Convergence was achieved for all analyses at 20 000 “burn in” runs and 30 000 model runs. A random-effects model with informative priors was used to best minimize the consequences of the diversity of the assorted patient populations and designs for each study.

Clinical postoperative outcomes were examined by calculating the pooled estimates of odds ratios (ORs) and 95% CIs of direct comparisons between any 2 MBP and OAB approaches. Direct evidence and indirect evidence for all MBP and OAB approaches were combined to estimate the examined outcomes, with a 95% equal-tail credible interval (CrI). We used NetMetaXL for rank probabilities to be plotted against the possible ranks for a treatment to result in the production of a graphical rankogram. This method of visually representing probabilities was combined with a surface under the cumulative ranking line for each surgical intervention.

**Figure 1.**
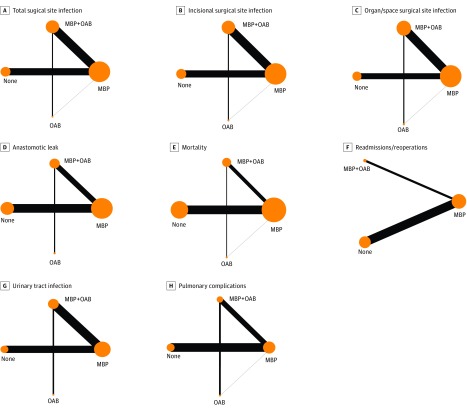
Network Plots of the 8 Outcomes Showing Direct Comparisons and Indirect Comparisons Between Treatment Groups Based on Mechanical Bowel Preparation (MBP) and Oral Antibiotic (OAB) Status Comparison networks were explored by representing each of the 4 bowel preparation approaches as a node, with lines between nodes representing a comparison between 2 linked treatments. Size of the node is proportional to the number of patients randomized to that bowel preparation approach, and the thickness of the lines is proportional to the number of studies comparing the 2 approaches.

We explored the comparison network by representing each of the 4 bowel preparation approaches as a node, with lines between nodes representing a comparison between 2 linked treatments. We considered the distribution of effect modifiers to be the same in all of the pairwise comparisons according to the transitivity assumption. Where there were inconsistencies between direct and indirect evidence, we evaluated clinical and methodological variables to identify possible causes of inconsistency (Table). Publication bias was assessed with funnel plots (eFigure 2 in the Supplement). The network plots (Figure 1) and funnel plots were generated by the gemtc package in STATA (Stata MP, version 15; StataCorp LP).

**Table.** Characteristics of Included Studies and Randomized Patients^a^

Source	Study Period	Male, No.:Female, No.^b^	Treatment 1, Patients, No.	Treatment 2, Patients, No.	Oral Solution	Left, Right, or Mixed Location	Laparoscopic, Open, or Mixed Approach	Intravenous Antibiotic Type	Oral Antibiotic Type
**Randomized to MBP vs No Preparation**									
Ali,^17^ 2007	NA	NA	109	101	PEG	Mixed	Open	Ceftriaxone 2 g, metronidazole 1 g	NA
Bertani et al,^18^ 2011	2007-2010	65:49 60:55	114	115	PEG	Mixed	Mixed	Cefoxitin 2 g and then 1 g administered at 4, 12, and 24 h	NA
Bhat and Chakraborty,^19^ 2016	2012-2014	56:42 57:44	98	104	PEG	Mixed	Open	Ceftriaxone 1 g, metronidazole 500 mg, continued for 48 h	NA
Bhattacharjee et al,^20^ 2015	2010-2013	21:17 20:13	38	33	PEG	Mixed	Open	Cefuroxime 1.5 g, metronidazole 500 mg, 1 h before surgery	NA
Bretagnol et al,^21^ 2010	2007-2009	56:33 46:43	89	89	Senna, povidone-iodine enema	Left	Mixed	Ceftriaxone1 g, metronidazole 500 mg, continued every 2 h during the surgical procedure	NA
Bucher et al,^22^ 2005	2001-2003	47:31 34:41	78	75	PEG	Left	Open	Ceftriaxone 1 g, metronidazole 500 mg, continued for at least 24 h	NA
Burke et al,^23^ 1994	1988-1992	52:30 43:44	82	87	Sodium picosulfate	Left	Open	Ceftriaxone 1 g, metronidazole 500 mg, then metronidazole 500 mg administered at 8 h and 16 h	NA
Contant et al,^24^ 2007	1998-2004	337:333 345:339	670	684	PEG, bisacodyl or sodium phosphate	Mixed	Open	As per institution guidelines	NA
Fa-Si-Oen et al,^25^ 2005	1998-2002	58:67 56:69	125	125	PEG	Mixed	Open	Cephazolin 2 g, metronidazole 1.5 g or gentamicin 240 mg, metronidazole 1.5 g	NA
Khan et al,^26^ 2011	NA	NA	51	51	PEG	Mixed	Open	Ceftriaxone 2 g, metronidazole 1 g	NA
Miettinen et al,^27^ 2000	1994-1996	68:70 62:67	138	129	PEG	Mixed	Open	Ceftriaxone 2 g, metronidazole 1 g	NA
Pena-Soria et al,^28^ 2008	2001-2007	35:29 33:22	65	64	PEG	Mixed	Open	Gentamicin 80 mg, metronidazole 500 mg, repeat dose at 8, 16, and 24 h	NA
Platell et al,^29^ 2006	2000-2005	NA	147	147	PEG	Mixed	Open	Ticarcillin-clavulanate 3.1 g or gentamicin 2 mg/kg, metronidazole 500 mg	NA
Ram et al,^30^ 2005	1999-2002	99:65 102:63	164	165	Sodium phosphate	Mixed	Open	Ceftriaxone 1 g, metronidazole 500 mg, continued for 48 h	NA
Saha et al,^31^ 2014	2008-2010	NA	32	31	PEG	Left	Open	Ceftriaxone 1 g, metronidazole 500 mg, continued for 36 h	NA
Santos et al,^32^ 1994	1991-1992	NA	72	77	Laxative, mannitol	Mixed	Open	Cephalothin 1 g, metronidazole 500 mg, then cephalothin 1 g given at 6 h and 12 h and metronidazole 500 mg at 8 h and 16 h	NA
Sasaki et al,^33^ 2012	2009	17:21 24:17	38	41	PEG	Mixed	Mixed (laparoscopic data given)	Flomoxef 1 g, continued every 3 h during surgery	NA
**Randomized to MBP With OAB vs OAB**									
Reddy et al,^34^ 2007	NA	22:20 11:11	42	22	Sodium picosulfate, magnesium citrate	Mixed	Open	NA	Neomycin 1 g, 3 doses 1 d before surgery, with or without synbiotic
Zmora et al,^11^ 2003	1997-2000	103:84 94:99	187	193	PEG	Mixed	Open	Intravenous antibiotics, type not specified, at induction of anesthesia and continued at least 24 h	Neomycin, erythromycin, 3 doses before surgery
Zmora et al,^12^ 2006	1997-2001	67:53 65:64	120	129	PEG	Left	Open	Metronidazole 500 mg, gentamicin 240 mg, ampicillin 1 g	Neomycin 1 g, erythromycin 1 g, 3 doses 1 d before surgery
**Randomized to MBP With OAB vs MBP**									
Beggs et al,^35^ 1982	NA	25:21 26:25	46	51	As per surgeon	Mixed	Open	Metronidazole 500 mg, then continued every 8 h for a further 5 doses	Metronidazole 200 mg for 4 d before surgery (all patients received neomycin 1 g orally 4 times daily for 5 d before surgery)
Dion et al,^36^ 1980	NA	NA	39	39	Magnesium citrate	Mixed	Open	Metronidazole 1 g, then 500 mg at 8 h and 16 h	Metronidazole 750 mg 3 times daily for 2 d before surgery (all patients received 1 g of neomycin 3 times daily 1 d before surgery)
Espin-Basany et al,^37^ 2005	NA	130:70 62:38	200	100	Sodium phosphate	Mixed	Open	Cefoxitin 1 g, then continued 1 g at 8 h and 16 h	Neomycin 1 g, metronidazole 1 g, either 3 doses or 1 dose 1 d before surgery
Hata et al,^38^ 2016	2007-2012	153:136 175:115	289	290	Picosulfate, magnesium citrate	Mixed	Laparoscopic	Metronidazole 750 mg, cefmetazole 1 g, then every 3 h during surgery	Kanamycin 1 g, metronidazole 750 mg, 2 doses 1 d before surgery
Ikeda et al,^39^ 2016	2013-2014	142:113 141:115	255	256	Picosulfate, magnesium citrate	Mixed	Laparoscopic	Cefmetazole 1 g, then every 3 h during surgery, continued for 24 h	Kanamycin 1 g, metronidazole 750 mg, 2 doses 1 d before surgery
Ishida et al,^40^ 2001	1998-2000	47:25 42:29	72	71	PEG	Mixed	Open	Cefotiam 1 g, then 1 g at completion of surgery, then 1 g twice daily for 2 d (6 doses in total)	Kanamycin 2 g/d, erythromycin 1.6 g/d, 4 doses for 2 d before surgery
Kling and Dahlgren,^41^ 1989	1985-1986	14:13 11:16	27	27	Bisacodyl, magnesium sulfate	Mixed	Open	Metronidazole 1.5 g, ceftriaxone 2 g	Neomycin 1 g, erythromycin 1 g, 3 doses 1 d before surgery
Kobayashi et al,^42^ 2007	2001-2004	154:88 137:105	242	242	PEG	Mixed	Open	Cefmetazole 1 g, then every 3 h during surgery, continued daily for 72 h	Kanamycin 1 g, erythromycin 400 mg, 3 doses 1 d before surgery
Lau et al,^43^ 1988	1981-1987	1.3:1 1.3:1	65	67	Bisacodyl, magnesium sulfate	Mixed	Open	Metronidazole 500 mg, gentamicin 2 mg/kg, then repeated at 8 h and 16 h	Neomycin 1 g, erythromycin 1 g, 3 doses 1 d before surgery
Lazorthes et al,^44^ 1982	1979-1980	20:10 14:16	30	30	Magnesium sulfate	Mixed	Open	Cephradine 2 g, metronidazole 500 mg, with or without gentamicin 2 mg/kg	Kanamycin 1 g, metronidazole 250 mg, 4 doses daily for 3 d before surgery
Lewis,^45^ 2002	1992-1995	NA	109	106	Sodium phosphate	Mixed	Open	Amikacin 1 g, metronidazole 1 g	Neomycin 2 g, metronidazole 2 g, 2 doses 1 d before surgery
Oshima et al,^46^ 2013	2006-2009	55:42 57:41	97	98	Magnesium citrate	Mixed	Open	Flomoxef, then every 3 h during surgery	Kanamycin 500 mg, metronidazole 500 mg, 3 doses 1 d before surgery
Playforth et al,^47^ 1988	NA	31:30 32:26	61	58	Mannitol	Mixed	Open	Metronidazole 500 mg	Neomycin 1 g every 6 h, metronidazole 200 mg every 8 h, 1 d before surgery
Raahave et al,^48^ 1988	NA	21:29 24:26	50	50	Bisacodyl, magnesium sulfate	Mixed	Open	Cefotaxime 2 g, then 2 g at 6 h and 12 h	Neomycin 1 g, erythromycin 1 g, 3 doses 1 d before surgery (ampicillin 2 g powdered in wound at closure)
Reddy et al,^34^ 2007	NA	22:20 11:13	42	24	Picosulfate, magnesium citrate	Mixed	Open	NA	Neomycin 1 g, 3 doses 1 d before surgery, with or without synbiotic
Sadahiro et al,^49^ 2014	2008-2011	49:51 51:44	100	95	Picosulfate, PEG	Mixed	Mixed	Flomoxef 1 g	Kanamycin 500 mg, metronidazole 500 mg, 3 doses 1 d before surgery
Stellato et al,^50^ 1990	1987	NA	38	45	Picosulfate, PEG	Mixed	Open	Cefoxitin	Neomycin, erythromycin
Weaver et al,^51^ 1986	NA	NA	29	31	As per surgeon	Mixed	Open	Ceftriaxone 2 g, metronidazole 1.5 g	Neomycin 1 g, erythromycin 1 g, 3 doses 1 d before surgery
Yabata et al,^52^ 1997	NA	23:17 29:22	40	51	PEG	Mixed	Open	Cefmetazole 1 g, then every 3 h during surgery	Tobramycin 30 mg, metronidazole 250 mg, 3 doses daily for 3 d before surgery (tobramycin 30 mg with saline instilled into lumen during surgery)
**Randomized to MBP vs OAB**									
Reddy et al,^34^ 2007	NA	11:13 11:11	24	22	Picosulfate, magnesium citrate	Mixed	Open	NA	Neomycin 1 g, 3 doses 1 d before surgery, with or without synbiotic

Abbreviations: MBP, mechanical bowel preparation; NA, not applicable; OAB, oral antibiotics; PEG, polyethylene glycol.

^a^ The study by Reddy et al^34^ reports on MBP with OAB, MBP only, and OAB only.

^b^ Sex breakdown for the fourth and fifth columns.

**Figure 2.**
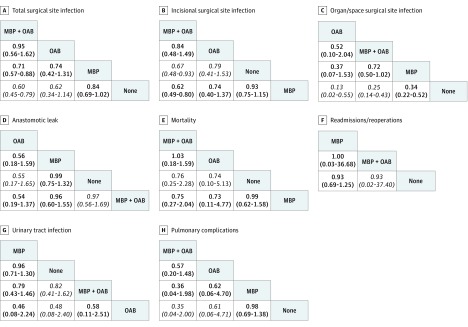
League Tables of the 8 Outcomes Showing Direct Comparisons and Indirect Comparisons Between Treatment Groups Based on Mechanical Bowel Preparation (MBP) and Oral Antibiotic (OAB) Status Outcomes are shown as odds ratios (95% equal-tail credible intervals); direct comparisons are represented in bold, and indirect comparisons are represented in italics.

**Figure 3.**
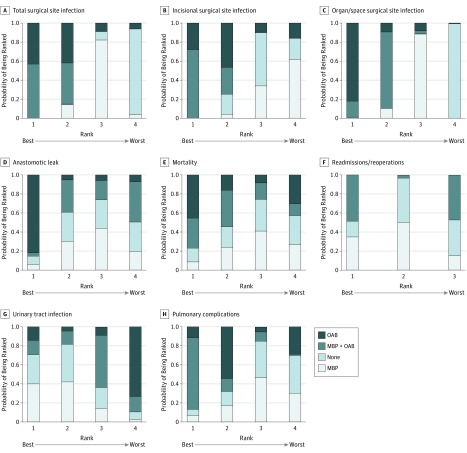
Rankograms of the 8 Outcomes Showing the Probability of Being Ranked the Best vs the Worst Based on Mechanical Bowel Preparation (MBP) and Oral Antibiotic (OAB) Status Outcomes on the far left of the x-axis are ranked best; far right, worst.

**Figure 4.**
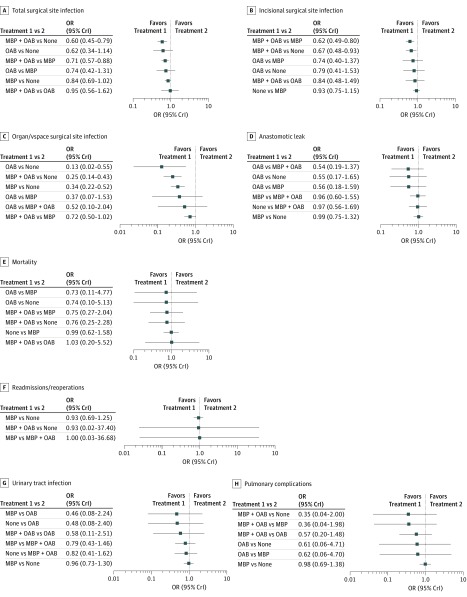
Forest Plots of the 8 Outcomes Showing Direct Comparisons and Indirect Comparisons Based on Mechanical Bowel Preparation (MBP) and Oral Antibiotic (OAB) Status Outcomes are shown as odds ratios (ORs) (95% equal-tail credible intervals [CrIs]).

All results are presented as relative effects and Bayesian estimates of the probability of each technique being the best to the worst relating to every studied outcome using rankograms (Figure 2), league tables (Figure 3), and forest plots (Figure 4).

## Results

A total of 1198 studies were identified through 5 electronic database searches and from other sources, such as reference lists. After applying inclusion and exclusion criteria and removal of duplicate studies, there were 17 studies^17,18,19,20,21,22,23,24,25,26,27,28,29,30,31,32,33,53,54^ identified comparing MBP vs no preparation (2117 vs 2128 patients), 3 studies^11,12,34^ identified comparing MBP with OAB vs OAB (349 vs 344 patients), and 19 studies^34,35,36,37,38,39,40,41,42,43,44,45,46,47,48,49,50,51,52^ identified comparing MBP with OAB vs MBP (1831 vs 1731 patients). In total, 38 RCTs among 8458 patients (52.1% male) were included. Features of included studies are summarized in the Table. Raw results reported in studies for SSI, anastomotic leak, and mortality are listed in eTables 2, 3, and 4 in the Supplement.

Most studies in this NMA used IVAB as part of the routine protocol, regardless of type of MBP and OAB approach. However, several studies compared OAB with IVAB (ie, patients receiving OAB with or without MBP did not receive IVAB). These trials included studies by Beggs et al,^35^ Dion et al,^36^ Kling and Dahlgren,^41^ Raahave et al,^48^ Reddy et al,^34^ and Weaver et al.^51^ Furthermore, several studies had 2 separate groups of patients who received OAB with and without IVAB. These trials included studies by Lau et al,^43^ Lazorthes et al,^44^ and Stellato et al.^50^

Most studies used a combination of either ceftriaxone, cefoxitin, cefuroxime, flomoxef, cephazolin, amikacin with or without metronidazole with or without gentamicin, or ticarcillin-clavulanate as the IVAB of choice. Two studies^11,12^ using IV ticarcillin-clavulanate were excluded because their comparison OAB group was tinidazole. The OAB regimens used were most commonly neomycin with or without erythromycin (9 studies), followed by kanamycin or neomycin with metronidazole (6 studies) (the Table lists the specific OAB used in each study). Among most studies in which OAB was used, at least 3 doses of OAB were prescribed for a duration of 1 to 3 days before surgery.

Most studies reported outcomes for combined right and left procedures except for 5 studies^12,21,22,23,31^ that reported on left-sided outcomes. Most studies used an open approach to CRS except for 2 studies^38,39^ using a laparoscopic approach and 4 studies^18,21,33,49^ reporting on data from a mix of open and laparoscopic procedures. Quality appraisal of included RCTs is summarized in eTable 1 in the Supplement.

There were 4 direct comparisons (MBP with OAB vs MBP, MBP with OAB vs OAB, MBP vs no preparation, and MBP vs OAB) and 2 indirect comparisons (MBP with OAB vs no preparation and OAB vs no preparation) based on the transitivity assumption (if A > B and B > C, then A > C). Figure 1 shows the network plots for direct comparisons and indirect comparisons. Direct comparisons have been boldfaced on the rankograms and league tables, and indirect comparisons have been italicized.

### Total SSI

Our NMA demonstrated a statistically significant reduction in total SSI for MBP with OAB compared with MBP only (OR, 0.71; 95% CrI, 0.57-0.88). Indirectly, there was also a risk reduction in SSI for MBP with OAB compared with no preparation (OR, 0.60; 95% CrI, 0.45-0.79). There was no difference in SSI between MBP only vs no preparation (OR, 0.84; 95% CrI, 0.69-1.02).

There was no significant difference in SSI between MBP with OAB vs OAB only (OR, 0.95; 95% CrI, 0.56-1.62). However, on Bayesian rankogram analysis, MBP with OAB was associated with a higher probability of the lowest total SSI rate after surgery than OAB only.

The reduction in SSI rate for OAB compared with MBP was not statistically significant (OR, 0.74; 95% CrI, 0.42-1.31). Similarly, the reduction in SSI rate for OAB compared with no preparation was not statistically significant (OR, 0.62; 95% CrI, 0.34-1.14).

The rankogram analysis, league tables, and forest plots all indicated that MBP with OAB had the highest probability of having the lowest total postoperative SSI rate, followed by OAB only, MBP only, and no preparation. These results are shown in Figures 2, 3, and 4.

### Incisional SSI

From the NMA model, MBP with OAB had a statistically significant lower rate of incisional SSI compared with MBP only (OR, 0.62; 95% CrI, 0.49-0.80) and no preparation (OR, 0.67; 95% CrI, 0.48-0.93). The difference in incisional SSI rate with MBP with OAB vs OAB only was not statistically significant (OR, 0.84; 95% CrI, 0.48-1.49). The difference in SSI rate between OAB only vs MBP only was not statistically significant (OR, 0.74; 95% CrI, 0.40-1.37). Similarly, the difference in incisional SSI rate between OAB vs no preparation was not statistically significant (OR, 0.79; 95% CrI, 0.41-1.53).

Both the rankogram analysis and the league tables demonstrated that MBP with OAB had the highest probability of having the lowest incisional SSI rate, followed by OAB only, no preparation, and MBP only. These results are shown in Figures 2 and 3.

### Organ/Space SSI

The NMA model revealed a statistically significant reduction in organ/space SSI for OAB only (OR, 0.13; 95% CrI, 0.02-0.55), MBP with OAB (OR, 0.25; 95% CrI, 0.25-0.43), and MBP only (OR, 0.34; 95% CrI, 0.22-0.52) over no preparation, respectively. It is important to note that OAB vs no preparation and MBP with OAB vs no preparation were indirect comparisons.

The difference in organ/space SSI rate between MBP with OAB vs MBP only did not reach statistical significance (OR, 0.72; 95% CrI, 0.50-1.02), but this analysis included studies in which patients were randomized to OAB without IVAB. When we excluded studies reporting on OAB without IVAB, the result reached statistical significance. The difference between OAB only and MBP with OAB did not reach statistical significance, with a wide 95% CrI (OR, 0.52; 95% CrI, 0.10-2.04).

Based on rankogram results, OAB only had the highest probability of having the lowest postoperative organ/space SSI rate, followed by MBP with OAB, MBP only, and no preparation. This NMA clearly showed that no preparation was associated with a statistically significant increase in organ/space SSI compared with the other 3 groups.

### Anastomotic Leak

Our NMA was unable to demonstrate a statistically significant difference in anastomotic leak rates between any of the 4 approaches. For MBP with OAB, this NMA did not show a risk reduction in anastomotic leaks compared with MBP only (OR, 0.96; 95% CrI, 0.60-1.55) and compared with no preparation (OR, 0.97; 95% CrI, 0.56-1.69). There was also no risk reduction in anastomotic leaks for MBP only vs no preparation (OR, 0.99; 95% CrI, 0.75-1.32). None of the following comparisons between OAB only and other approaches reached statistical significance: OAB only vs MBP with OAB (OR, 0.54; 95% CrI, 0.19-1.37), OAB only vs MBP only (OR, 0.56; 95% CrI, 0.18-1.59), and OAB only vs no preparation (OR, 0.55; 95% CrI, 0.17-1.65). The rankogram analysis and league tables also demonstrated that OAB only had the highest probability of having the lowest rate of postoperative anastomotic leak. However, the small numbers in the OAB only group are a caveat to this result. There was virtually no difference in anastomotic leak rates between the other 3 approaches on NMA and Bayesian Monte Carlo rankogram.

### Mortality

There was no difference in perioperative mortality between the 4 approaches from the NMA. Comparing MBP with OAB vs OAB only, no difference was observed (OR, 1.03; 95% CrI, 0.20-5.52). The difference between MBP with OAB and OAB only compared with both MBP only and no preparation was not statistically significant. The wide 95% CrI when comparing all groups was likely associated with the low perioperative mortality rate in all groups, and it is difficult to draw valid conclusions in terms of perioperative mortality. There was no difference in the perioperative mortality rate, with a narrow 95% CrI between MBP only and no preparation (OR, 0.99; 95% CrI, 0.62-1.58).

### Readmissions/Reoperations

There was no difference in readmissions/reoperations between the groups. Insufficient data were obtained to compare OAB only with other approaches in terms of readmissions/reoperations. For MBP with OAB vs MBP only, the OR was 1.00 (95% CrI, 0.03-36.68). For MBP with OAB vs no preparation, the OR was 0.93 (95% CrI, 0.02-37.40). Although there was no difference between these approaches, the wide 95% CrI suggests that the data are inconclusive. No difference was found between MBP only and no preparation, with a narrow 95% CrI (OR, 0.93; 95% CrI, 0.69-1.25).

### Urinary Tract Infection

The NMA demonstrated no significant difference in UTI rates between the 4 approaches. The strategies of MBP with OAB and OAB only did not reduce UTI rates after surgery. When MBP with OAB was compared with OAB only, MBP with OAB was associated with lower rates of UTI, although the 95% CrI was wide and did not reach statistical significance (OR, 0.58; 95% CrI, 0.11-2.51). Because of the wide 95% CrI, it is difficult to draw accurate conclusions from the NMA between the groups in terms of UTI rates.

### Pulmonary Complications

The NMA demonstrated no significant differences in terms of pulmonary complications, including aspiration pneumonia, between the 4 approaches. Although the 95% CrIs were wide when comparing OAB only and no preparation (OR, 0.61; 95% CrI, 0.06-4.71) and when comparing OAB only and MBP only (OR, 0.62; 95% CrI, 0.06-4.70), the rankogram analysis and league tables strongly ranked MBP with OAB as the best approach to reduce pulmonary complications. The rate of pulmonary complications when comparing MBP with OAB vs OAB only was not statistically significant (OR, 0.57; 95% CrI, 0.20-1.48). The difference in pulmonary complications between MBP with OAB vs MBP only was also not statistically significant (OR, 0.36; 95% CrI, 0.04-1.98). In addition, the indirect comparison between MBP with OAB vs no preparation was not statistically significant (OR, 0.35; 95% CrI, 0.04-2.00).

## Discussion

This NMA provided 4 direct comparisons (MBP with OAB vs MBP, MBP with OAB vs OAB, MBP vs no preparation, and MBP vs OAB) and 2 indirect comparisons (MBP with OAB vs no preparation and OAB vs no preparation) of MBP and OAB approaches before elective CRS, with the following 8 short-term outcomes evaluated: total SSI, incisional SSI, organ/space SSI, anastomotic leak, mortality, readmissions/reoperations, UTI, and pulmonary complications. Like other meta-analyses^4,5,6,55,56,57^ on this topic, most of the RCTs included in this study reported on outcomes based on open CRS.

The 2 studies included in this NMA that reported data based on laparoscopic cohorts had different findings. Hata et al^38^ found a reduced risk of SSI (5.5%) associated with MBP with OAB vs MBP. However, Ikeda et al^39^ found no difference in infectious complications between MBP with OAB vs MBP (5.9% for both).

This NMA identified only 5 RCTs^12,21,22,23,31^ reporting on left-sided CRS. The study by Bretagnol et al^21^ showed that there was a higher risk of infectious complications without MBP before elective rectal cancer sphincter-saving surgery. In contrast, Bucher et al^22^ reported increased morbidity with MBP, and both Saha et al^31^ and Burke et al^23^ reported no difference in anastomotic leak rate with and without MBP.

The results of our Bayesian analysis support the findings of a recent meta-analysis of RCTs by Chen et al^5^ that compared MBP with OAB vs MBP and showed that MBP with OAB was associated with reduced infectious complications. This finding is also similar to conclusions drawn from recent, large population-based American College of Surgeons–National Surgical Quality Improvement Program (ACS-NSQIP) studies^58,59,60,61^ and the Cochrane reviews by Nelson et al.^7,8^ Furthermore, our study reported findings similar to other meta-analyses,^4,6^ including the Cochrane review by Güenaga et al^4^ comparing MBP vs no preparation, which showed no difference between the 2 approaches in terms of mortality, anastomotic leak, SSI, and reoperation. However, our NMA was not able to replicate the increased risk of harm with MBP reported in the meta-analyses by Slim et al^55^ and by Bucher et al.^57^

To clarify the most important network findings of our NMA, the rest of this section focuses mainly on 3 direct comparisons reported in this NMA (MBP vs no preparation, MBP with OAB vs OAB, and MBP with OAB vs MBP) and characterizes the limitations and strengths of the studies included, as well as the results from the Bayesian analysis. Because there was limited direct comparison between MBP vs OAB and because comparisons between MBP with OAB vs no preparation and OAB vs no preparation were indirect comparisons made on the transitivity assumption, the results reported for these comparisons were not based on strong evidence and represent gaps in the literature.

### MBP vs No Preparation (17 Studies) Among 4245 Patients

Most studies^17,18,21,22,23,24,25,26,27,28,29,30,31,32,33,53,54^ included in this NMA reported no difference in infectious complications when comparing MBP vs no preparation.^17,18,23,25,27,28,30,31,33,53,54^ In contrast, Bretagnol et al,^21^ Contant et al,^24^ and Platell et al^29^ reported a lower risk of infectious complications with MBP. Bucher et al^22^ and Santos et al^32^ reported increased morbidity and infectious complications, respectively, with MBP. Most RCTs were single-center trials. Studies ranged from a single-surgeon trial^28^ to multicenter trials.^21,24,25^ Intravenous antibiotics were administered at induction of anesthesia in all trials comparing MBP vs no preparation. A broad-spectrum cephalosporin with and without metronidazole was the most common regimen, followed by gentamicin and metronidazole. In the multicenter trial reported by Contant et al,^24^ the choice of IVAB prophylaxis regimen was according to the guideline for prevention of SSI issued by the infectious diseases department at each participating hospital.

Of the studies that reported better outcomes with MBP vs no preparation, Bretagnol et al^21^ reported specifically on elective rectal cancer sphincter-preserving surgery from the French Research Group of Rectal Cancer Surgery (GRECCAR) III RCT. The main advantages were decreased infectious complications after anastomotic leakage reported by Contant et al^24^ and decreased risk of anastomotic leakage requiring reoperation reported by Platell et al.^29^ In contrast, the study by Bucher et al^22^ that reported on elective left-sided CRS found increased risk of morbidity with MBP, and the study by Santos et al^32^ reported increased risk of wound infection with MBP vs no preparation (24% vs 12%) but no difference in risk of anastomotic leak.

On Bayesian analysis, our NMA was unable to demonstrate a statistically significant benefit of MBP vs no preparation for most of the outcome measures except for a reduction in the rate of organ/space SSI. There was no significant reduction in total SSI, incisional SSI, anastomotic leak, perioperative mortality, readmission rates, UTI, or pulmonary complications. These outcomes are consistent with most of the RCTs analyzed in this NMA and with existing meta-analyses^12,21,24,28,62,63,64^ that have shown no benefit associated with MBP in the context of CRS. Our NMA is consistent with the findings from the 2011 Cochrane review^4^ (18 RCTs among 5805 patients) that demonstrated no significant reduction in anastomotic leak rates or wound infections with MBP over no preparation. The conclusion of the meta-analysis by Dahabreh et al^6^ (18 RCTs, 7 nonrandomized comparative studies, and 6 single-group cohorts) was that MBP was similar to no preparation with respect to mortality, anastomotic leakage, and wound infection. Our NMA confirmed no benefit of MBP vs no preparation on Bayesian analysis.

### MBP With OAB vs OAB (3 Studies) Among 693 Patients

Only 3 RCTs^11,12,34^ comparing MBP with OAB vs OAB were included in this NMA. These comprise the study by Reddy et al^34^ and 2 studies by Zmora et al.^11,12^ Zmora et al^12^ in 2006 reported on left-sided CRS. In 2003, Zmora et al^11^ reported on both right-sided and left-sided colon and rectal surgery. While the 2003 study^11^ included more patients, the 2006 study^12^ provided important data on left-sided CRS. While other overlapping studies were excluded, the 2006 study^12^ was included for qualitative analysis, and including and excluding that study for quantitative synthesis did not change the outcome of the Bayesian analysis. In the study by Reddy et al,^34^ patients were randomized to the following 4 groups: MBP only, neomycin plus MBP, synbiotics plus neomycin plus MBP, and synbiotics plus neomycin but no MBP. None of the 3 studies in this subsection showed a statistically significant difference between MBP with OAB vs OAB. The results of the study by Reddy et al^34^ suggested that synbiotics plus neomycin plus MBP reduced fecal Enterobacteriaceae and bacterial translocation, but there was no clinically relevant difference when comparing the 4 groups.

On Bayesian analysis, none of the differences in outcome measures reached statistical significance. The rankogram analysis and league tables ranked MBP with OAB better than OAB in terms of total SSI, incisional SSI, UTI, and pulmonary complications. In terms of organ/space SSI and anastomotic leak, OAB was ranked better than MBP with OAB. Compared with no preparation, MBP with OAB, MBP, and OAB were associated with a statistically significant reduction in organ/space SSI. There was no difference on the rankogram between MBP with OAB and OAB in terms of mortality and readmissions/reoperations. The difficulty in deciding between MBP with OAB and OAB has been demonstrated by the lack of consensus between ACS-NSQIP studies. While 2 recent studies^59,65^ have shown that MBP with OAB is associated with the lowest risk of infectious complications, other ACS-NSQIP studies^66,67^ have recommended OAB on the basis that addition of MBP with OAB provided no additional benefit.

This NMA was not able to show a statistically significant difference between MBP with OAB and OAB. That group was small, and the direct comparison between MBP with OAB and OAB was limited. However, MBP with OAB consistently ranked as the best regimen in 4 of 8 outcome measures and as second best in 2 of the 8 outcome measures. In 2 of the 8 outcome measures, OAB ranked best, and OAB was associated with a statistically significant reduction in organ/space SSI. Neither MBP with OAB nor OAB reduced the rate of UTI.

### MBP With OAB vs MBP (19 Studies) Among 3562 Patients

Nineteen studies^34,35,36,37,38,39,40,41,42,43,44,45,46,47,48,49,50,51,52^ comparing MBP with OAB vs MBP were included in this NMA. In this group, there was methodological diversity. A range of IVAB and OAB for varying durations was used in these RCTs. Intravenous antibiotics administered in RCTs included metronidazole, cephalosporins, combination cephalosporin with metronidazole, and amikacin with metronidazole. Oral antibiotics included metronidazole, neomycin, kanamycin, kanamycin with erythromycin, neomycin with erythromycin, kanamycin with metronidazole, neomycin with metronidazole, and tobramycin with metronidazole. Studies used different doses of antibiotics for various durations before surgery.

While most studies used IVAB at induction of anesthesia (regardless of OAB status), the studies by Beggs et al,^35^ Dion et al,^36^ Kling and Dahlgren,^41^ Raahave et al,^48^ Reddy et al,^34^ and Weaver et al^51^ compared OAB with IVAB. In these studies, the OAB group did not receive IVAB. In the studies by Beggs et al^35^ and by Dion et al,^36^ all patients received oral neomycin, and patients were randomized to IV metronidazole vs oral metronidazole. In the studies by Weaver et al^51^ and Kling and Dahlgren,^41^ patients were randomized to either IV ceftriaxone and metronidazole or oral neomycin and erythromycin.^51^ The study by Raahave et al^48^ compared IV cefotaxime with oral neomycin-erythromycin. As stated earlier, Reddy et al^34^ randomized patients into the following 4 groups: MBP only, oral neomycin plus MBP, synbiotics plus neomycin plus MBP, and synbiotics plus neomycin without MBP. In these studies,^35,36,41,48,51^ there was no benefit reported for OAB over IVAB, and the study by Weaver et al^51^ was discontinued after 60 patients were enrolled because of the high rate of infection (41%) in the oral neomycin and erythromycin group. As already mentioned, the study by Reddy et al^34^ showed no reduction in SSI with oral neomycin but reported reduced fecal Enterobacteriaceae and bacterial translocation in the MBP, oral neomycin, and synbiotic groups. Furthermore, a subgroup of patients receiving OAB without IVAB in the studies by Lau et al,^43^ Lazorthes et al,^44^ and Stellato et al^50^ had increased risk of septic complications. In the study by Lazorthes et al,^44^ the rates of septic complications for patients receiving either oral kanamycin and metronidazole vs IV cephradine and metronidazole vs both oral and IVAB were 30%, 23%, and 3.3%, respectively. In the study by Lau et al,^43^ the rates of postoperative septic complications in patients receiving oral neomycin and erythromycin vs IV gentamicin and metronidazole vs both were 27.4%, 11.9%, and 12.3%, respectively. Comparing oral neomycin and erythromycin only vs IV cefoxitin only vs both OAB and IVAB, Stellato et al^50^ reported rates of postoperative septic complications of 11.4%, 11.7%, and 7.8%, respectively.

In contrast, a large number of studies^38,40,44,45,46,47,49^ comparing MBP with OAB vs MBP reported benefit of MBP with OAB over MBP in reducing SSI. In studies reporting a benefit of MBP with OAB, it is important to note that OAB was given in addition to IVAB at induction of anesthesia rather than as a replacement for IVAB. In summary, studies in which MBP with OAB was administered without IVAB showed no benefit in terms of infectious complications over MBP.

On Bayesian analysis, this NMA showed a statistically significant improvement in total SSI and incisional SSI when MBP with OAB was used compared with MBP. For organ/space SSI, the OR was 0.72 (95% CrI, 0.50-1.02). There was no statistically significant difference in rates of mortality, UTI, and pulmonary complications.

### Limitations and Strengths

Our study had several limitations. First, there were only 2 studies^38,39^ reporting specifically on a laparoscopic approach and 4 studies^18,21,33,49^ reporting on a combination of open and laparoscopic techniques, and surgery in most studies was performed with an open approach. This does not reflect the current status in which most colorectal surgical procedures are performed laparoscopically. Second, we were not able to report outcomes based on colon vs rectum, side of resection, and formation of stoma because of a lack of available data. Third, there were only 3 studies^4,44,45^ comparing MBP with OAB vs OAB (among a total of 693 patients), and this may have led to failure to detect statistically significant differences between MBP with OAB and OAB. Fourth, there were variations in IVAB and OAB, as well as type of MBP used (most studies used ethylene glycol or sodium phosphate as the preferred agent). There were also variations in the dosage, duration of antibiotics used before surgery, and duration continued after surgery. Fifth, several of the comparisons in this NMA were indirect based on the transitivity assumption rather than actual data from RCTs. For this reason, as stated earlier, we boldfaced the direct comparisons in the rankogram analysis and league tables and italicized the indirect comparisons to easily distinguish direct and indirect comparisons.

Despite these limitations, our study has several key strengths. First, by using the network NMA, we were able to combine large quantitative evidence from RCTs to simultaneously compare MBP with OAB, MBP, OAB, and no preparation, which would be unlikely to be addressed by an RCT with 4 arms. Second, we used Bayesian Monte Carlo modeling to rank the 4 preparation approaches using both a rankogram analysis and league tables, providing a simple visual representation of which technique is better for each outcome measure. Third, we included only RCTs to reduce the risk of bias and ensure standardization and the validity of the results, and we appraised quality of studies by the Cochrane Collaboration risk of bias tool. Fourth, our results are consistent with the results of the Cochrane review^4^ comparing MBP vs no preparation and with the ACS-NSQIP studies^59,60^ on MBP with OAB and OAB. Furthermore, our results draw together the findings of previous meta-analyses^4,5,6^ comparing different approaches of MBP and OAB regimens into a comprehensive network study with clear graphical visualization of how each approach compares and ranks with one another. The next step may be a full factorial design with all 4 approaches included in the same trial, which would be the ideal way to address the issue at hand.

## Conclusions

This NMA demonstrated that MBP with OAB is associated with the lowest risk of SSI in patients undergoing elective CRS. Oral antibiotics only may be beneficial, but there was insufficient evidence in the existing literature to draw conclusive results. In this NMA, MBP with OAB was ranked as the best approach for most of the outcome measures evaluated herein. In terms of SSI, there was no difference between MBP and no preparation before elective CRS. Both MBP and no preparation were associated with increased SSI compared with MBP with OAB and OAB. Evidence from RCTs comparing MBP with OAB vs OAB is limited, and further research should concentrate on increasing this evidence base. The long-standing practice of MBP and no preparation is still a common approach and should be revisited after careful reconsideration of the growing body of evidence from RCTs and population-based studies.

## Notes

Supplement. **eFigure 1.** PRISMA Flow Diagram **eFigure 2.** Funnel Plots **eAppendix.** Search Strategy, Selection Process and Data Extraction and Method of Network Analysis **eTable 1.** Cochrane Collaboration Risk of Bias Tool (Version 5.0.1): Risk of Bias in the Individual Randomized Controlled Trials Assessed by Cochrane Risk of Bias Checklist **eTable 2.** Presentation of Outcome Data for Total Surgical Site Infection, by Included Study **eTable 3.** Presentation of Outcome Data for Anastomotic Leak, by Included Study **eTable 4.** Presentation of Outcome Data for Mortality, by Included Study
