# Supplementary material for: Association of Mechanical Bowel Preparation and Oral Antibiotics Before Elective Colorectal Surgery With Surgical Site Infection: A Network Meta-analysis
Source: JAMA Netw Open. 2018 Oct 12;1(6):e183226. doi: 10.1001/jamanetworkopen.2018.3226 (PMC6324461; doi:10.1001/jamanetworkopen.2018.3226)
Supplement: Supplement. — eFigure 1. PRISMA Flow Diagram eFigure 2. Funnel Plots eAppendix. Search Strategy, Selection Process and Data Extraction and Method of Network Analysis eTable 1. Cochrane Collaboration Risk of Bias Tool (Version 5.0.1): Risk of Bias in the Individual Randomized Controlled Trials Assessed by Cochrane Risk of Bias Checklist eTable 2. Presentation of Outcome Data for Total Surgical Site Infection, by Included Study eTable 3. Presentation of Outcome Data for Anastomotic Leak, by Included Study eTable 4. Presentation of Outcome Data for Mortality, by Included Study [file jamanetwopen-1-e183226-s001.pdf]

Supplementary Online Content

Toh JWT, Phan K, Hitos K, et al. Association of mechanical bowel preparation and oral antibiotics before elective colorectal surgery with surgical site infection: a network meta-analysis. *JAMA Netw Open*. 2018;1(6):e183226. doi:10.1001/jamanetworkopen.2018.3226

eFigure 1. PRISMA Flow Diagram

eFigure 2. Funnel Plots

eAppendix. Search Strategy, Selection Process and Data Extraction and Method of Network Analysis

eTable 1. Cochrane Collaboration Risk-of-Bias Tool (Version 5.0.1): Risk of Bias in the Individual Randomized Controlled Trials Assessed by Cochrane Risk of Bias Checklist

eTable 2. Presentation of Outcome Data for Total Surgical Site Infection, by Included Study

eTable 3. Presentation of Outcome Data for Anastomotic Leak, by Included Study

eTable 4. Presentation of Outcome Data for Mortality, by Included Study

This supplementary material has been provided by the authors to give readers additional information about their work.

© 2018 Toh JWT et al. *JAMA Network Open*.

eFigure 1. PRISMA Flow Diagram

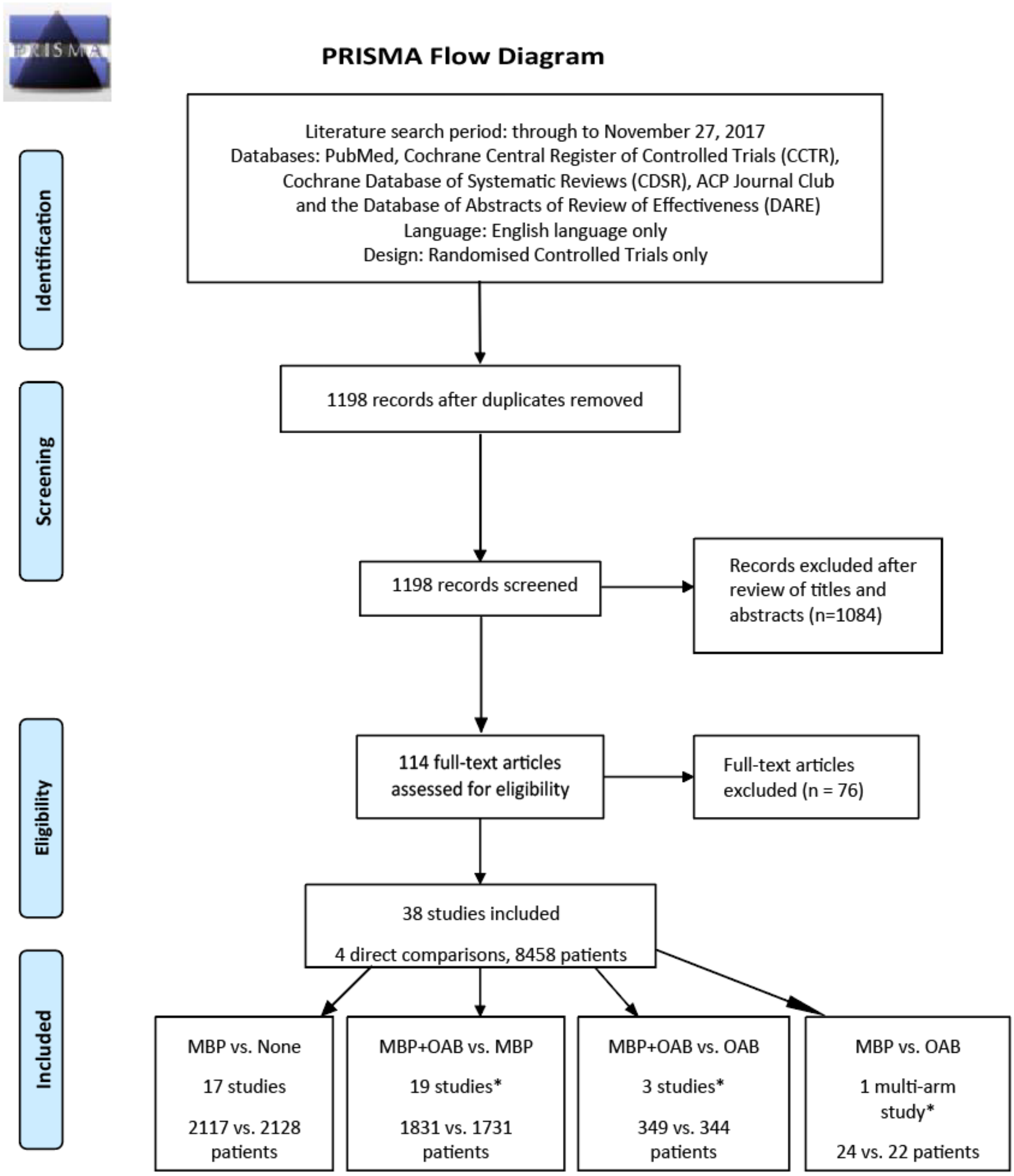

From: Moher D, Liberati A, Tetzlaff J, Altman DG, The PRISMA Group (2009). *P*referred *R*eporting *I*tems for *S*ystematic Reviews and *M*eta-*A*nalyses: The PRISMA Statement. PLoS Med 6(6): e1000097. doi:10.1371/journal.pmed1000097

* Multi-arm study comparing three different bowel preparation approaches

eFigure 2. Funnel Plots

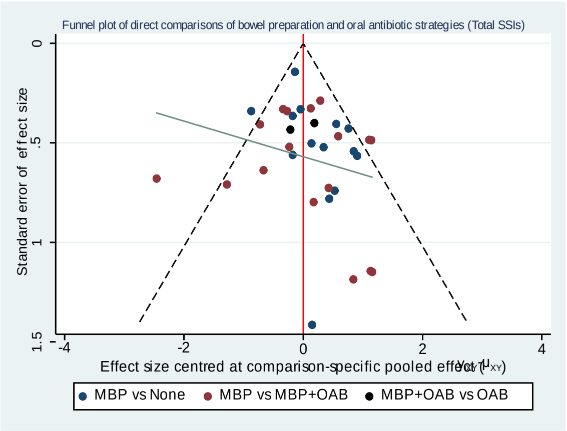

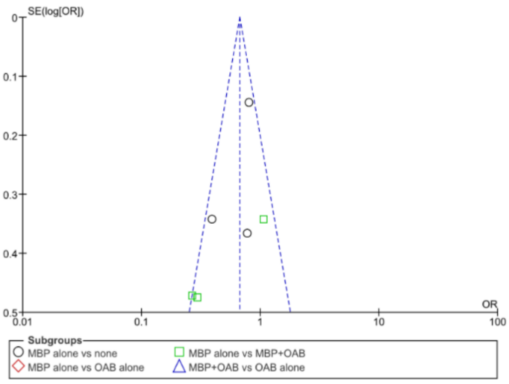

Funnel plot of direct comparisons of bowel preparation and oral antibiotic strategies (laparoscopic / mixed studies only) (Total SSI)

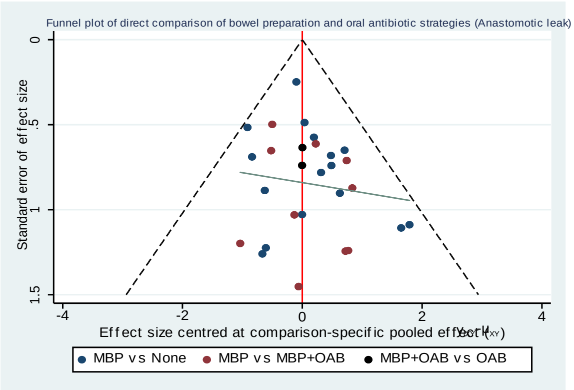

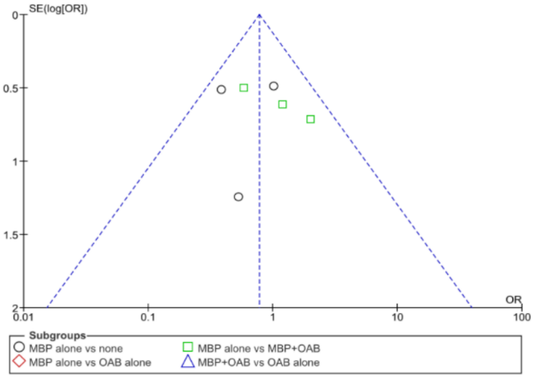

Funnel plot of direct comparisons of bowel preparation and oral antibiotic strategies (laparoscopic / mixed studies only) (Anastomotic leak)

© 2018 Toh JWT et al. *JAMA Network Open*.

eAppendix. Search Strategy, Selection Process and Data Extraction and Method of Network Analysis

Search Strategy

We performed a comprehensive literature according to the Preferred Reporting Items for Systematic Reviews and Meta-Analysis (PRISMA) guidelines.[1,2] Five electronic databases were searched including PubMed, Cochrane Central Register of Controlled Trials (CCTR), Cochrane Database of Systematic Reviews (CDSR), ACP Journal Club and the Database of Abstracts of Review of Effectiveness (DARE) from database inception to current (November 27, 2017). To minimise the risk of overlooking relevant studies and given the wide variety of procedural nomenclature, it was necessary to combine a large number of key words and MeSH terms. This constituted the terms “mechanical bowel preparation”, “oral antibiotics”, “colon”, “rectal”, “colorectal”, “surgery”. We also used ClinicalTrials.gov to identify recently completed trials and PROSPERO to identify recently completed systematic reviews.

Number	Search
1	colon.mp. [mp=ti, ot, ab, tx, kw, ct, sh, hw, nm, fx, kf, px, rx, an, ui, sy]
2	rectal.mp. [mp=ti, ot, ab, tx, kw, ct, sh, hw, nm, fx, kf, px, rx, an, ui, sy]
3	colorectal.mp. [mp=ti, ot, ab, tx, kw, ct, sh, hw, nm, fx, kf, px, rx, an, ui, sy]
4	surgery.mp. [mp=ti, ot, ab, tx, kw, ct, sh, hw, nm, fx, kf, px, rx, an, ui, sy]
5	mechanical bowel preparation.mp. [mp=ti, ot, ab, tx, kw, ct, sh, hw, nm, fx, kf, px, rx, an, ui, sy]
6	antibiotic.mp. [mp=ti, ot, ab, tx, kw, ct, sh, hw, nm, fx, kf, px, rx, an, ui, sy]
7	(1 or 2 or 3) and 4 and (5 or 6)
8	Duplicates removed

Selection process and data extraction

Two independent reviewers assessed the eligibility of each study for inclusion, with any disagreements being resolved by review and consensus. The following data were extracted for each study: author, year of publication, study period, journal, study design, status of mechanical bowel preparation and oral antibiotics, bowel preparation used, right vs. left side of resection, laparoscopic/open/mixed cohort, type of intravenous antibiotics used and type of

oral antibiotics used. The data for primary and secondary outcomes were then extracted for each study.

Network Meta-analysis

We conducted a network meta-analysis using Bayesian Markov Chain Monte Caro (MCMC) method in WinBUGS 1.4.3 (MRC Biostatistics Unit, Cambridge, UK) through the conduit of the Microsoft Excel based macro NetMetaXL 1.6.1 (Canadian Agency for Drugs and Technologies in Health).[3] A convergence test for each analysis was conducted by checking whether the Monte Carlo error was less than 5% of the standard deviation of the effect estimates or the variance between the studies. Convergence was achieved for all analyses at 20,000 “burn in” runs and 30,000 model runs. A random effects model with informative priors was used to best minimise the impact of the diversity of the assorted patient populations and designs for each study.

Clinical post-operative outcomes were examined calculating the pooled estimates of odds ratios (OR) and 95% confidence interval (CI) of direct comparisons between any 2 of bowel preparation and oral antibiotic strategies. Direct and indirect evidence for all MBP and OAB strategies were combined to estimate the examined outcomes, with a 95% equal tail credible interval (CrI). Bayesian analysis was implemented because of its ability to simultaneously compare multiple treatment options. We used NetMetaXL in order for rank probabilities to be plotted against the possible ranks for a treatment to result in the production of a graphical “rankogram”. This method of visually representing probabilities was combined with a surface under the cumulative ranking line for each surgical intervention (SUCRA). For example, a SUCRA of 0.5 means that there is a 50% chance that the respective intervention is the best option in achieving the lowest rate of an undesirable clinical outcome.

© 2018 Toh JWT et al. *JAMA Network Open*.

All results were presented as relative effects and Bayesian estimates of the probability of each technique being the best to the worst relating to every studied outcome with rankograms, league tables and forest plots.

1. Moher D, Liberati A, Tetzlaff J, Altman DG. Preferred reporting items for systematic reviews and meta-analyses: the PRISMA statement. *PLoS medicine.* 2009;6(7):e1000097.
2. Phan K, Tian DH, Cao C, Black D, Yan TD. Systematic review and meta-analysis: techniques and a guide for the academic surgeon. *Annals of cardiothoracic surgery.* 2015;4(2):112-122.
3. Turner RM, Davey J, Clarke MJ, Thompson SG, Higgins JP. Predicting the extent of heterogeneity in meta-analysis, using empirical data from the Cochrane Database of Systematic Reviews. *International journal of epidemiology.* 2012;41(3):818-827.

© 2018 Toh JWT et al. *JAMA Network Open*.

eTable 1. Cochrane Collaboration Risk-of-Bias Tool (Version 5.0.1): Risk of Bias in the Individual Randomized Controlled Trials Assessed by Cochrane Risk of Bias Checklist

First author	Random sequence allocation	Allocation concealment	Blinding of participants	Blinding of outcome assessment	Incomplete outcomes data
**MBP+ OAB- versus MBP- OAB-**					
Ali[1]	Unclear	Unclear	High	High	Unclear
Bertani[2]	Low	Unclear	High	High	Unclear
Bhat[3]	Low	Low	Low	High	Low
Bhattacharjee[4]	Low	Low	High	High	Unclear
Bretagnol[5]	Low	Low	Low	High	Low
Bucher[6]	Low	Low	Low	High	Low
Burke[7]	Low	Unclear	Low	Unclear	Low
Contant[8]	Low	Low	Low	Low	Low
Fa-Si-Oen[9]	Low	Low	Unclear	Unclear	Low
Khan[10]	Unclear	Unclear	High	High	Unclear
Miettinen[11]	Unclear	Unclear	High	High	Unclear
Pena-Soria[12]	Low	Unclear	Low	High	Low
Platell[13]	Low	Low	Unclear	Unclear	Low
Ram[14]	Unclear	High	Unclear	Unclear	Low
Saha[15]	High	High	High	High	High
Santos[16]	Unclear	High	High	High	High
Sasaki[17]	Unclear	Unclear	High	High	High
**MBP+ OAB+ vs MBP- OAB+**					
Reddy[18]	Low	Unclear	High	High	Low
Zmora (2003)[19]	Low	High	High	High	High
Zmora (2006)[20]	Low	High	High	High	High
**MBP+OAB+ vs MBP+OAB-**					
Beggs[21]	Unclear	High	Low	High	High
Dion[22]	Low	Low	Low	Low	High
Espin-Basany[23]	Unclear	Unclear	High	Low	Low
Hata[24]	Low	Low	High	High	Low
Ikeda[25]	Unclear	Unclear	High	Low	Low
Ishida[26]	Low	Low	High	High	Low
Kling[27]	Unclear	High	High	High	High
Kobayashi[28]	Low	High	High	High	Low
Lau[29]	Low	High	High	Low	Low
Lazorthes[30]	Unclear	High	High	High	Low
Lewis[31]	Low	Low	Low	Low	Low
Oshima[32]	Low	High	High	Low	High
Playforth[33]	Unclear	High	High	High	Low

© 2018 Toh JWT et al. *JAMA Network Open*.

Raahave[34]	Low	Low	High	High	Low
Reddy[18]	Low	Unclear	High	High	Low
Sadahiro[35]	Low	Low	High	Unclear	Low
Stellato[36]	Low	High	Low	Low	High
Weaver[37]	Unclear	High	High	High	Low
Yabata[38]	Low	Unclear	High	High	High
**MBP+ OAB- vs MBP- OAB+**					
Reddy[18]	Low	Unclear	High	High	Low

REFERENCES

1. Ali M. Randomized prospective clinical trial of no preparation versus mechanical bowel preparation before elective colorectal surgery. Med Channel. 2007;13:32–35.

2. Bertani E, Chiappa A, Biffi R, et al. Comparison of oral polyethylene glycol plus a large volume glycerine enema with a large volume glycerine enema alone in patients undergoing colorectal surgery for malignancy: a randomized clinical trial. Colorectal disease : the official journal of the Association of Coloproctology of Great Britain and Ireland. 2011;13(10):e327-334.

3. Bhat AP, Fazl; Chowdri, Nisar; Wani, Rauf; Thakur, Natasha; Nazki, Saalim; Wani, Imtiaz. Mechanical bowel preparation versus no preparation in elective colorectal surgery: A prospective randomized study. International Journal of Surgery Open. 2016;2:26-30.

4. Bhattacharjee PKC, Saibal Chakraborty. An Open-Label Prospective Randomized Controlled Trial of Mechanical Bowel Preparation vs Nonmechanical Bowel Preparation in Elective Colorectal Surgery: Personal Experience. Indian J Surg. 2015;77 (Suppl 3)(S1233-S1236).

5. Bretagnol F, Panis Y, Rullier E, et al. Rectal cancer surgery with or without bowel preparation: The French GRECCAR III multicenter single-blinded randomized trial. Ann Surg. 2010;252(5):863-868.

6. Bucher P, Gervaz P, Soravia C, Mermillod B, Erne M, Morel P. Randomized clinical trial of mechanical bowel preparation versus no preparation before elective left-sided colorectal surgery. The British journal of surgery. 2005;92(4):409-414.

7. Burke P, Mealy K, Gillen P, Joyce W, Traynor O, Hyland J. Requirement for bowel preparation in colorectal surgery. The British journal of surgery. 1994;81(6):907-910.

8. Contant CM, Hop WC, van't Sant HP, et al. Mechanical bowel preparation for elective colorectal surgery: a multicentre randomised trial. Lancet (London, England). 2007;370(9605):2112-2117.

9. Fa-Si-Oen P, Roumen R, Buitenweg J, et al. Mechanical bowel preparation or not? Outcome of a multicenter, randomized trial in elective open colon surgery. Dis Colon Rectum. 2005;48(8):1509-1516.

10. Khan S, Hadi A, Ahmad S, Shah F, Iqbal Z, Khan M. Mechanical bowel preparation in elective colorectal surgery. J Med Sci. 2007;19:31-34.

© 2018 Toh JWT et al. *JAMA Network Open*.

11. Miettinen RP, Laitinen ST, Makela JT, Paakkonen ME. Bowel preparation with oral polyethylene glycol electrolyte solution vs. no preparation in elective open colorectal surgery: prospective, randomized study. Dis Colon Rectum. 2000;43(5):669-675; discussion 675-667.

12. Pena-Soria MJ, Mayol JM, Anula R, Arbeo-Escolar A, Fernandez-Represa JA. Single-blinded randomized trial of mechanical bowel preparation for colon surgery with primary intraperitoneal anastomosis. J Gastrointest Surg. 2008;12(12):2103-2108; discussion 2108-2109.

13. Platell C, Barwood N, Makin G. Randomized clinical trial of bowel preparation with a single phosphate enema or polyethylene glycol before elective colorectal surgery. The British journal of surgery. 2006;93(4):427-433.

14. Ram E, Sherman Y, Weil R, Vishne T, Kravarusic D, Dreznik Z. Is mechanical bowel preparation mandatory for elective colon surgery? A prospective randomized study. Archives of surgery (Chicago, Ill : 1960). 2005;140(3):285-288.

15. Saha AK, Chowdhury F, Jha AK, Chatterjee S, Das A, Banu P. Mechanical bowel preparation versus no preparation before colorectal surgery: A randomized prospective trial in a tertiary care institute. Journal of natural science, biology, and medicine. 2014;5(2):421-424.

16. Santos JC, Jr., Batista J, Sirimarco MT, Guimaraes AS, Levy CE. Prospective randomized trial of mechanical bowel preparation in patients undergoing elective colorectal surgery. The British journal of surgery. 1994;81(11):1673-1676.

17. Sasaki J, Matsumoto S, Kan H, et al. Objective assessment of postoperative gastrointestinal motility in elective colonic resection using a radiopaque marker provides an evidence for the abandonment of preoperative mechanical bowel preparation. Journal of Nippon Medical School = Nippon Ika Daigaku zasshi. 2012;79(4):259-266.

18. Reddy BS, Macfie J, Gatt M, Larsen CN, Jensen SS, Leser TD. Randomized clinical trial of effect of synbiotics, neomycin and mechanical bowel preparation on intestinal barrier function in patients undergoing colectomy. The British journal of surgery. 2007;94(5):546-554.

19. Zmora O, Mahajna A, Bar-Zakai B, et al. Colon and rectal surgery without mechanical bowel preparation: a randomized prospective trial. Ann Surg. 2003;237(3):363-367.

20. Zmora O, Mahajna A, Bar-Zakai B, et al. Is mechanical bowel preparation mandatory for left-sided colonic anastomosis? Results of a prospective randomized trial. Tech Coloproctol. 2006;10(2):131-135.

21. Beggs FD, Jobanputra RS, Holmes JT. A comparison of intravenous and oral metronidazole as prophylactic in colorectal surgery. The British journal of surgery. 1982;69(4):226-227.

22. Dion YM, Richards GK, Prentis JJ, Hinchey EJ. The influence of oral versus parenteral preoperative metronidazole on sepsis following colon surgery. Ann Surg. 1980;192(2):221-226.

23. Espin-Basany E, Sanchez-Garcia JL, Lopez-Cano M, et al. Prospective, randomised study on antibiotic prophylaxis in colorectal surgery. Is it really necessary to use oral antibiotics? Int J Colorectal Dis. 2005;20(6):542-546.

© 2018 Toh JWT et al. *JAMA Network Open*.

24. Hata H, Yamaguchi T, Hasegawa S, et al. Oral and Parenteral Versus Parenteral Antibiotic Prophylaxis in Elective Laparoscopic Colorectal Surgery (JMTO PREV 07-01): A Phase 3, Multicenter, Open-label, Randomized Trial. Ann Surg. 2016;263(6):1085-1091.

25. Ikeda A, Konishi T, Ueno M, et al. Randomized clinical trial of oral and intravenous versus intravenous antibiotic prophylaxis for laparoscopic colorectal resection. The British journal of surgery. 2016;103(12):1608-1615.

26. Ishida H, Yokoyama M, Nakada H, Inokuma S, Hashimoto D. Impact of oral antimicrobial prophylaxis on surgical site infection and methicillin-resistant Staphylococcus aureus infection after elective colorectal surgery. Results of a prospective randomized trial. Surgery today. 2001;31(11):979-983.

27. Kling PA, Dahlgren S. Oral prophylaxis with neomycin and erythromycin in colorectal surgery. More proof for efficacy than failure. Archives of surgery (Chicago, Ill : 1960). 1989;124(6):705-707.

28. Kobayashi M, Mohri Y, Tonouchi H, Miki C, Nakai K, Kusunoki M. Randomized clinical trial comparing intravenous antimicrobial prophylaxis alone with oral and intravenous antimicrobial prophylaxis for the prevention of a surgical site infection in colorectal cancer surgery. Surgery today. 2007;37(5):383-388.

29. Lau WY, Chu KW, Poon GP, Ho KK. Prophylactic antibiotics in elective colorectal surgery. The British journal of surgery. 1988;75(8):782-785.

30. Lazorthes F, Legrand G, Monrozies X, et al. Comparison between oral and systemic antibiotics and their combined use for the prevention of complications in colorectal surgery. Dis Colon Rectum. 1982;25(4):309-311.

31. Lewis RT. Oral versus systemic antibiotic prophylaxis in elective colon surgery: a randomized study and meta-analysis send a message from the 1990s. Canadian journal of surgery Journal canadien de chirurgie. 2002;45(3):173-180.

32. Oshima T, Takesue Y, Ikeuchi H, et al. Preoperative oral antibiotics and intravenous antimicrobial prophylaxis reduce the incidence of surgical site infections in patients with ulcerative colitis undergoing IPAA. Dis Colon Rectum. 2013;56(10):1149-1155.

33. Playforth MJ, Smith GM, Evans M, Pollock AV. Antimicrobial bowel preparation. Oral, parenteral, or both? Dis Colon Rectum. 1988;31(2):90-93.

34. Raahave D, Hesselfeldt P, Pedersen TB. Cefotaxime i.v. versus oral neomycin-erythromycin for prophylaxis of infections after colorectal operations. World J Surg. 1988;12(3):369-373.

35. Sadahiro S, Suzuki T, Tanaka A, et al. Comparison between oral antibiotics and probiotics as bowel preparation for elective colon cancer surgery to prevent infection: prospective randomized trial. Surgery. 2014;155(3):493-503.

36. Stellato TA, Danziger LH, Gordon N, et al. Antibiotics in elective colon surgery. A randomized trial of oral, systemic, and oral/systemic antibiotics for prophylaxis. Am Surg. 1990;56(4):251-254.

37. Weaver M, Burdon DW, Youngs DJ, Keighley MR. Oral neomycin and erythromycin compared with single-dose systemic metronidazole and ceftriaxone prophylaxis in elective colorectal surgery. Am J Surg. 1986;151(4):437-442.

© 2018 Toh JWT et al. *JAMA Network Open*.

38. Yabata E, Okabe S, Endo M. A prospective, randomized clinical trial of preoperative bowel preparation for elective colorectal surgery--comparison among oral, systemic, and intraoperative luminal antibacterial preparations. Journal of medical and dental sciences. 1997;44(4):75-80.

© 2018 Toh JWT et al. *JAMA Network Open*.

eTable 2. Presentation of Outcome Data for Total Surgical Site Infection, by Included Study

Direct comparison of mechanical bowel preparation and oral antibiotic strategies

Study	MBP			None			MBP+OAB			OAB		
	Events, n	Patients, n		Events, n	Patients, n		Events, n	Patients, n		Events, n	Patients, n	
Bertani	16	114	14%	20	115	17%						
Bhat	6	98	6%	8	104	8%						
Bhattacharje e	14	38	37%	7	33	21%						
Bretagnol	18	89	20%	35	89	39%						
Bucher	11	78	14%	5	75	7%						
Burke	4	82	5%	3	87	3%						
Contant	105	670	16%	128	684	19%						
Fa-Si-Oen	9	125	7%	7	125	6%						
Miettinen	5	138	4%	3	129	2%						
Pena-Soria	19	65	29%	11	64	17%						
Platell	20	147	14%	22	147	15%						
Ram	17	164	10%	11	165	7%						
Saha	16	32	50%	15	31	48%						
Sasaki	1	38	3%	1	41	2%						
Reddy	5	24	21%				6	42	14%	3	22	14%
Zmora (2003)							14	187	7%	13	193	7%
Zmora (2006)							10	120	8%	14	129	11%
Beggs	5	51	10%				6	46	13%			
Dion	4	39	10%				1	39	3%			
Espin-Basany	10	100	10%				22	200	11%			
Hata	37	290	13%				21	289	7%			
Ikeda	20	256	8%				20	255	8%			
Ishida	17	71	24%				8	72	11%			
Kling	3	27	11%				1	27	4%			
Kobayashi	26	242	11%				17	242	7%			

© 2018 Toh JWT et al. *JAMA Network Open*.

Lau	9	67	13%				8	65	12%			
Lazorthes	4	30	13%				1	30	3%			
Lewis	21	106	20%				6	109	6%			
Oshima	22	98	22%				6	97	6%			
Raahave	3	50	6%				7	50	14%			
Sadahiro	22	95	23%				22	100	22%			
Stellato	7	45	16%				3	38	8%			
Weaver	4	31	13%				16	29	55%			

MBP – mechanical bowel preparation; OAB – oral antibiotics

© 2018 Toh JWT et al. *JAMA Network Open*.

eTable 3. Presentation of Outcome Data for Anastomotic Leak, by Included Study

Direct comparison of mechanical bowel preparation and oral antibiotic strategies												
Study	MBP			None			MBP+OAB			OAB		
	Events, n	Patients, n		Events, n	Patients, n		Events, n	Patients, n		Events, n	Patients, n	
Ali	6	109	6%	1	101	1%						
Bertani	9	114	8%	9	115	8%						
Bhat	2	98	2%	4	104	4%						
Bhattacharjee	4	38	11%	2	33	6%						
Bretagnol	6	89	7%	14	89	16%						
Bucher	5	78	6%	1	75	1%						
Contant	32	670	5%	37	684	5%						
Fa-Si-Oen	7	125	6%	6	125	5%						
Khan	6	51	12%	4	51	8%						
Miettinen	5	138	4%	3	129	2%						
Pena-Soria	4	65	6%	3	64	5%						
Platell	3	147	2%	7	147	5%						
Ram	1	164	1%	2	165	1%						
Saha	2	32	6%	2	31	6%						
Santos	7	72	10%	4	77	5%						
Sasaki	1	38	3%	2	41	5%						
Zmora (2003)							7	187	4%	4	193	2%
Zmora (2006)							5	120	4%	3	129	2%
Beggs	1	51	2%				1	46	2%			
Hata	6	290	2%				5	289	2%			
Ikeda	6	256	2%				3	255	1%			
Ishida	2	71	3%				1	72	1%			
Lau	2	67	3%				1	65	2%			

© 2018 Toh JWT et al. *JAMA Network Open*.

Lewis	1	106	1%				3	109	3%			
Playforth	4	58	7%				7	61	11%			
Raahave	5	40	13%				2	33	6%			
Sadahiro	7	95	7%				12	100	12%			
Stellato	2	45	4%				2	38	5%			

MBP – mechanical bowel preparation; OAB – oral antibiotics

© 2018 Toh JWT et al. *JAMA Network Open*.

eTable 4. Presentation of Outcome Data for Mortality, by Included Study

Direct comparison of mechanical bowel preparation and oral antibiotic strategies												
Study	MBP			None			MBP+OAB			OAB		
	Events, n	Patients, n		Events, n	Patients, n		Events, n	Patients, n		Events, n	Patients, n	
Ali	6	109	6%	1	101	1%						
Bhat	0	98	0%	0	104	0%						
Bhattacharjee	1	38	3%	1	33	3%						
Bretagnol	1	89	1%	0	89	0%						
Bucher	0	78	0%	0	75	0%						
Burke	2	82	2%	0	87	0%						
Contant	20	670	3%	26	684	4%						
Fa-Si-Oen	2	125	2%	1	125	1%						
Miettinen	0	138	0%	0	129	0%						
Pena-Soria	3	65	5%	4	64	6%						
Platell	4	147	3%	1	147	1%						
Ram	2	164	1%	2	165	1%						
Santos	0	72	0%	0	77	0%						
Reddy	0	24	0%				0	42	0%	0	22	0%
Zmora (2003)							3	187	2%	3	193	2%
Ikeda	0	256	0%				0	255	0%			
Lazorthes	1	30	3%				1	30	3%			
Playforth	5	58	9%				6	61	10%			
Raahave	3	50	6%				0	50	0%			

MBP – mechanical bowel preparation; OAB – oral antibiotics

© 2018 Toh JWT et al. *JAMA Network Open*.
